# Host-protein biomarkers distinguish asymptomatic TB in an active case finding study

**DOI:** 10.5588/ijtldopen.25.0203

**Published:** 2025-09-10

**Authors:** J.L. Pedersen, J. Ho, H.C. Lai, N.J. Bokil, T.A. Nguyen, P.T.B. Nguyen, G.J. Fox, G.B. Marks, W.J. Britton, B.M. Saunders

**Affiliations:** ^1^ School of Life Sciences, University of Technology Sydney, Australia;; ^2^ Centenary Institute, TB Research Program, Sydney, New South Wales, Australia;; ^3^ Woolcock Institute of Medical Research, Sydney, NSW, Australia;; ^4^ James Cook University, Australia;; ^5^ Woolcock Institute of Medical Research, Hanoi Vietnam;; ^6^ The University of Sydney Vietnam Institute, Ho Chi Minh City, Vietnam;; ^7^ WHO Collaborating Centre for Tuberculosis and the Sydney Infectious Diseases Institute (Sydney ID), The University of Sydney, Sydney, NSW, Australia;; ^8^ South Western Sydney Clinical School, University of New South Wales, Australia;; ^9^ Burnet Institute, Melbourne, Australia.

**Keywords:** tuberculosis, plasma biomarkers, diagnosis, subclinical asymptomatic disease

Dear Editor,

Active TB disease is primarily identified following presentation of symptomatic individuals and diagnosis by sputum screening. However, recent studies have highlighted that up to 50% of individuals with active TB are asymptomatic, or subclinical.^1^ These individuals are infectious, and contribute to the ongoing spread of TB within the community.^2^ It is therefore important to develop novel, non-sputum-based tests to identify all individuals with TB disease to reduce the global burden of TB. Circulating mRNA, microRNA and proteins, have all shown biomarker potential in this regard.^3–5^ Our group have previously identified a plasma protein signature that has biomarker potential, distinguishing symptomatic clinical TB patients from controls.^6^ The results presented here demonstrate the potential of this biomarker signature in an active case finding setting, to identify individuals with asymptomatic TB disease.

Participants were recruited from the Ca Mau Province of Southern Vietnam as part of a nested study within the active TB case finding study ACTRN12614000372684.^**3**^ Three cohorts were identified: individuals with clinical TB disease (treatment naïve), individuals with TB infection (TBI), and healthy controls. Clinical TB cases were defined by a positive GeneXpert result and either a positive sputum culture or chest x-ray consistent with active clinical TB disease. Individuals with a negative GeneXpert but a positive (QuantiFERON-TB Gold (Qiagen)) were diagnosed with TBI. Healthy controls were negative for both tests. Participant characteristics including age, sex and smoking status were described previously.^3^ Plasma from 85 individuals with TB disease, were compared with those from 75 individuals with TBI and 101 healthy controls.

TB disease was largely asymptomatic, and only identified through active case finding. Only 32 (37.9%), of those with active TB disease reported at least one symptom consistent with clinical TB, while similar number of those with TBI 30 (29.3%) and healthy controls 22 (29.7%) also reported at least one symptom consistent with clinical TB (cough on the day of screening, cough for the past two weeks or more, sputum production on the day of screening or sputum production for the past two weeks or more). Participants, all over the age of 15, provided a blood sample which was processed to plasma and stored at -80^o^C. Nine proteins were measured in the plasma samples: IP-10 (CXCL10), MCP-1 (CCL2), RANTES (CCL5), soluble TNF receptor 1 (sTNFR1), VEGF, Eotaxin, IL-6, IL-10 and TNF, by cytometric bead array (CBA; BD Biosciences, Australia) as per the manufacturer’s protocol. Samples below the limit of detection were allocated a value equal to the half limit of detection.

Ethical approval was obtained from the Human Research Ethics Committee of the University of Sydney, Australia and the Institutional Review Board of the National Lung Hospital, Hanoi, Vietnam under project number 2013/073, ‘Reducing the prevalence of TB in a highly endemic setting by community-wide active case finding’.

IP-10 and IL-6 were significantly elevated in individuals with clinical TB compared to both healthy controls and the TBI cohort (Figure). ROC curve analysis demonstrated that IP-10 was the most effective single marker, distinguishing clinical TB disease from the combined TBI- Healthy control groups with an AUC=0.708 and a specificity of 95.5%, but a sensitivity of only 32.9%. The diagnostic potential of the panel of all 9 proteins showed an AUC=0.739 to identify TB disease compared to the combined TBI and healthy control groups. Specificity was high at 94.9%, but sensitivity low at only 36.5%, with most of the diagnostic accuracy coming from the increase in IP-10 levels in TB patients. IP-10 has been identified as a promising biomarker for symptomatic TB disease in multiple studies.^7–9^ This study demonstrates, for the first time, that IP-10 can also identify active TB disease in asymptomatic or subclinical individuals. IP-10 has performed with high diagnostic accuracy in other TB biomarker studies, exhibiting an AUC range of 0.726–0.950.^7–10^ Additionally, IP-10 levels in plasma strongly correlated with risk of TB exposure in children, and when used in conjunction with an interferon-γ release assay, may improve diagnostic accuracy.^11^ IP-10 may be a useful initial triage tool. Plasma IP-10 levels over 1000 pg/mL identify 76.5% of TB patients, though with a high false positive rate in control subjects. The addition of IL-6 levels above 3000 fg/mL, would increase this accuracy to 88.2%. Applying these cut-offs in the previously described TB biomarker study, where patients were recruited following passive case detection, identifies 97% of TB patients, with a false positive rate of only 12% in the control cohort.^6^

**Figure.**
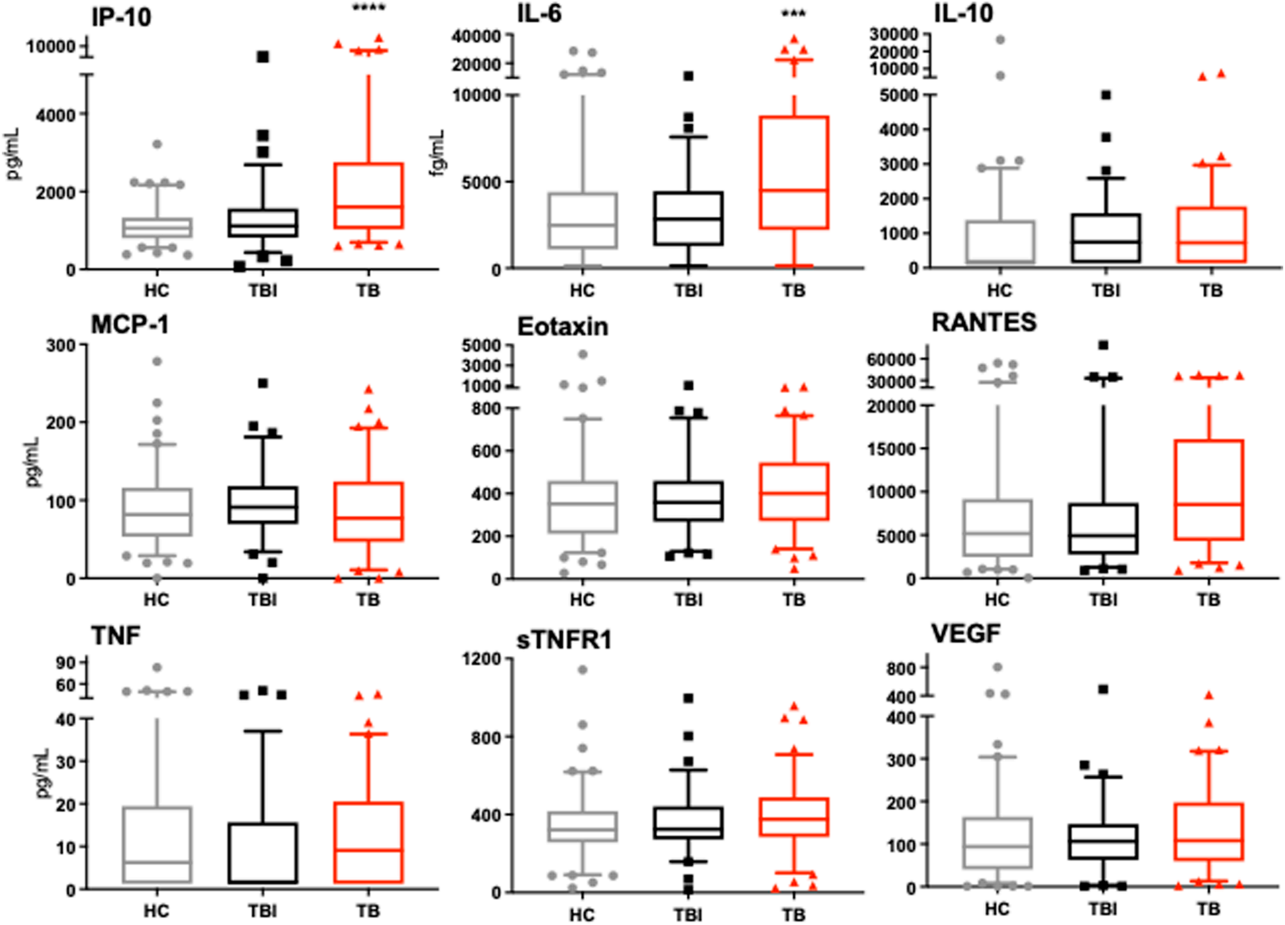
Expression of 9 plasma proteins in clinical TB patients (n=85) compared to individuals with TBI (n=75) and healthy controls (HC) (n=101). IP-10 and IL-6 levels in TB patients were significantly elevated in comparison to both TBI and HC cohorts (*** p-value<0.001, **** p-value<0.0001).

Stratification of the data for gender (males vs. females) and smoking status (smoker vs. non-smoker) identified no significant differences in protein expression between males and females of each group, with the exception of higher IL-10 levels in the female healthy controls compared to males (*p< 0.01*), and higher levels of RANTES (*p< 0.05*), and Eotaxin (*p< 0.01*), in smokers compared to non-smokers in the healthy control groups. Within the TB cohort, non-smokers had significantly elevated levels of IP-10 compared to smokers (*p< 0.01*).

Increasing the sensitivity of the biomarker signature, is a crucial constraint to reach the WHO’s target product profiles for non-sputum TB tests.^12^ Including additional protein analytes, mRNA or miRNA could be beneficial. The samples utilised in this study were collected as part of a mRNA biomarker profiling study, which found a 7 gene signature that could distinguish TB patients from control subjects with an AUC=86.0 and a sensitivity of 80.3%.^3^ This gene signature outperformed the nine-plasma protein biosignature, suggesting that measurable changes at the transcriptional level are present in early TB disease, and may enhance the sensitivity of protein biomarker candidates in a combined signature. Geographical, environmental and ethnic differences may also influence protein profiles, and these confounding factors will need to be considered in future studies. Our study found no differences in profiles of those with TBI and healthy controls, supporting the supposition that these proteins assessed are associated with the inflammation response to TB disease, rather than TBI.

In conclusion, our study indicates that IP-10 and IL-6 levels may serve as effective triage tools to identify TB disease, including in individuals with asymptomatic or subclinical TB. These data also highlight the need to consider all categorical states of TB, when developing new diagnostics for TB disease.
